# Treatment effect of DNA framework nucleic acids on diffuse microvascular endothelial cell injury after subarachnoid hemorrhage

**DOI:** 10.1111/cpr.13206

**Published:** 2022-02-21

**Authors:** Ruiqi Chen, Dingke Wen, Wei Fu, Lu Xing, Lu Ma, Yi Liu, Hao Li, Chao You, Yunfeng Lin

**Affiliations:** ^1^ 12530 Department of Neurosurgery West China Hospital Sichuan University Chengdu China; ^2^ Department of Gynecological Nursing West China Second University Hospital West China School of Nursing Key Laboratory of Birth Defects and Related Diseases of Women and Children (Ministry of Education) Sichuan University Chengdu China; ^3^ 12530 State Key Laboratory of Oral Diseases National Clinical Research Center for Oral Diseases West China Hospital of Stomatology Sichuan University Chengdu China; ^4^ 12530 College of Biomedical Engineering Sichuan University Chengdu China

**Keywords:** anti‐apoptosis, diffuse brain microvascular endothelial cells injury, PI3K‐AKT‐mTOR signaling pathways, subarachnoid hemorrhage, tetrahedral DNA nanostructures

## Abstract

**Objectives:**

The purpose of this study was to investigate the treatment effect and molecular mechanism of tetrahedral framework nucleic acids (tFNAs), novel self‐assembled nucleic acid nanomaterials, in diffuse BMEC injury after SAH.

**Materials and Methods:**

tFNAs were synthesized from four ssDNAs. The effects of tFNAs on SAH‐induced diffuse BMEC injury were explored by a cytotoxicity model induced by hemin, a breakdown product of hemoglobin, in vitro and a mouse model of SAH via internal carotid artery puncture in vivo. Cell viability assays, wound healing assays, transwell assays, and tube formation assays were performed to explore cellular function like angiogenesis.

**Results:**

In vitro cellular function assays demonstrated that tFNAs could alleviate hemin‐induced injury, promote angiogenesis, and inhibit apoptosis in hemin cytotoxicity model. In vivo study using H&E and TEM results jointly indicated that the tFNAs attenuate the damage caused by SAH in situ, showing restored number of BMECs in the endothelium layer and more tight intercellular connectivity. Histological examination of SAH model animals confirmed the results of the in vitro study, as tFNAs exhibited treatment effects against diffuse BMEC injury in the cerebral microvascular bed.

**Conclusions:**

Our study suggests the potential of tFNAs in ameliorating diffuse injury to BMECs after SAH, which laid theoretical foundation for the further study and use of these nucleic acid nanomaterials for tissue engineering vascularization.

## Graphical abstract

The potential of tFNAs in ameliorating diffuse injury to BMECs after SAH, laying a theoretical foundation for the further study and the use of these nucleic acid nanomaterials.

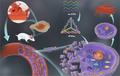

## 1 INTRODUCTION

Subarachnoid hemorrhage (SAH) is a devastating neurological disease and a major threat to health worldwide.^1^ Generally caused by intracranial aneurysm (IA) rupture, SAH is characterized by diffuse distribution of blood in the subarachnoid space, in which the cerebral microvascular bed is immersed in cerebrospinal fluid (CSF) containing cytotoxic metabolites of blood components.^2^, ^3^ The monolayer endothelial cells of the cerebral arterioles, also known as brain microvascular endothelial cells (BMECs), are vulnerable to injury due to the presence of anatomically weak vessel structures that lack elastic intima and smooth muscles in the media layer.^4^, ^5^ Studies have revealed that diffuse injury to the BMECs induced by cytotoxic metabolites of erythrocytes in the CSF is an important pathological change after SAH.^6^ As a trigger of “secondary brain injury” after SAH, diffuse BMEC injury is related to dysfunction of the BBB, an increased risk of cerebral vasospasm and delayed cerebral ischemia, which lead to poor prognosis in patients with SAH.^7^, ^8^ Unfortunately, effective treatments for diffuse BMEC injury after SAH are lacking.

In recent years, there has been rapid progress in the development of DNA nanomaterials for biomedical applications due to their excellent editability.^9^ Among these DNA nanomaterials, tetrahedral framework nucleic acids (tFNAs), which can be easily synthesized and edited, have become a major research focus owing to their 3D polyhedral structure and broad biological features.^10^ Extensive studies have revealed that tFNAs alone can regulate several cellular processes, such as migration, proliferation, differentiation, and autophagy.^11^, ^12^, ^13^ Our previous research reported that tFNAs have beneficial effects on normal endothelial cells under physiological conditions, regulating cell viability and promoting angiogenesis.^14^ Furthermore, abundant evidence has confirmed that tFNAs successfully alleviate apoptosis of various cell types by restoring normal nuclear morphology, reversing cell cycle abnormalities, and affecting apoptosis mediators.^12^, ^15^, ^16^

Therefore, it is important to investigate the treatment effect of tFNAs in SAH‐related diffuse BMEC injury in vitro and in vivo. The mechanism related to the antiapoptotic and proangiogenic functions of tFNAs was also explored. The results of our study may provide a novel aspect for the treatment of diffuse BMEC injury after SAH and lay a theoretical foundation for the further study and the use of tFNAs for tissue engineering vascularization.

## 2 MATERIALS AND METHODS

### 2.1 Cell culture

BMECs were acquired from Otwo Biotech (Catalog No: bEnd.3, HTX1926, Guangzhou, China). The cells were incubated in medium composed of 89% H‐DMEM (Gibco, NY, USA), 10% FBS, and 1% penicillin/streptomycin in plates or T25 flasks and cultured in a 37°C incubator with 95% air and 5% CO2.

### 2.2 Synthesis and characterization of tFNAs

The sequences of four single‐stranded DNAs (ssDNAs) are shown in Table 1. tFNAs were synthesized as previously reported.^16^ ssDNAs were added to TM buffer (consisting of 10 nmol L−1 Tris‐HCl (pH 8.0) and 50 nmol L−1 MgCl2) in equal proportions. The temperature of the system was initially elevated to 95°C for 10 min and then lowered to 4°C for 20 min. To confirm the successful synthesis of tFNAs, 8% polyacrylamide gel electrophoresis (PAGE) was conducted to determine the molecular weights of the tFNAs and ssDNAs. Transmission electron microscopy (TEM, Tecnai G2 F20 S‐TWIN, FEI, USA) and dynamic light scattering (DLS) were used to measure the particle size of the tFNA molecules. The zeta potential distribution of the tFNA molecules was measured with a Zetasizer Nano ZS90 instrument (Malvern Instrument Ltd., Malvern, UK).

**TABLE 1:** Base sequence of each single‐stranded DNA

ssDNA	Direction	Base sequence
S1	5′→3′	ATTTATCACCCGCCATAGTAGACGTATCACCAGGCAGTTGAGACGAACATTCCTAAGTCTGAA
S2	5′→3′	ACATGCGAGGGTCCAATACCGACGATTACAGCTTGCTACACGATTCAGACTTAGGAATGTTCG
S3	5′→3′	ACTACTATGGCGGGTGATAAAACGTGTAGCAAGCTGTAATCGACGGGAAGAGCATGCCCATCC
S4	5′→3′	ACGGTATTGGACCCTCGCATGACTCAACTGCCTGGTGATACGAGGATGGGCATGCTCTTCCCG

### 2.3 Uptake of tFNAs by BMECs

Flow cytometry and immunofluorescence staining were conducted to confirm the cellular uptake of tFNAs in BMECs and the influence on their biological behavior. BMECs were plated in 6‐well plates (2 × 10^6^ cells per well) and cultured overnight. The cells were then treated with Cy5‐modified tFNAs (250 nM) or Cy5‐modified ssDNAs (250 nM) for 12 h and analyzed by flow cytometry (Attune NxT, Thermo Fisher Scientific, MA, USA). The cytoskeleton was stained with phalloidin by immunofluorescence staining, and the nuclei were stained with 4,6‐diamidino‐2‐phenylindole (DAPI). Images were then obtained by confocal laser scanning microscopy (CLSM).

### 2.4 Cell viability assay

To evaluate the cytotoxic effect of hemin on BMECs, cells were plated in 96‐well plates (Corning) at a density of 1 × 10^4^ per well and cultured overnight to allow attachment. The cells were then exposed to hemin at various corresponding concentrations (30, 40, 50, 60, and 70 µM) for 12 h. Then, a Cell Counting Kit‐8 (CCK‐8, Dojindo, Japan) was used, and the absorbance of the samples was measured at 450 nm. The concentration of tFNAs (62.5, 125, 250, or 375 nM) that led to the highest cell viability was determined through the same approach. To examine the treatment effects of tFNAs against cytotoxicity induced by hemin, BMECs were divided into the following three groups: (i) the control group, (ii) the group treated with 50 µM hemin for 12 h, and (iii) the group preincubated with 50 µM hemin for 12 h and then treated with 250 nM tFNAs for 12 h. The samples were analyzed in the same way.

### 2.5 Wound healing assay

To explore the influence of tFNAs on BMEC migration, we conducted a wound healing experiment. BMECs were seeded in 6‐well plates and cultured for 24 h. Then, a scratch was made with a sterile pipette tip. The cells were divided into groups and treated as mentioned above, and wound images were taken 0, 12, and 24 h after incubation under a microscope (Olympus X71).

### 2.6 Transwell assay

To further evaluate the migration ability of BMECs, a transwell assay was performed using 24‐well transwell chambers with a pore size of 8 µm. Briefly, cells (4 × 10^5^ cells) were seeded in the upper chamber of the transwell insert. The cells were divided into groups and treated as mentioned above. BMECs that had attached to the bottom side of the membrane were fixed with 4% paraformaldehyde for 15 min and stained with 1% crystal violet for 20 min. Finally, the cells were counted under a microscope.

### 2.7 Tube formation assay

Tube formation experiment was performed to explore the influence of tFNAs on BMEC angiogenesis. Matrigel solution (50 μl per well) was added to a 96‐well plate (kept on ice) and incubated at 37°C for 1 h to allow gel formation. BMECs were cultured, divided into groups, and treated accordingly. After 24 h, 100 μl of BMECs (approximately 1.5 × 10^5^ cells/mL) were seeded in the prepared 96‐well plates (containing gels). Appropriate treatments (control, hemin, or hemin plus tFNAs) were added to the culture medium (without serum). After 12 h, tube formation was imaged. ImageJ software (ImageJ 1.53c) was used for statistical analysis.

### 2.8 Flow cytometry analysis of the cell cycle

Flow cytometry was used to analyze the cell cycle. BMECs were incubated, divided into groups as mentioned above, and treated accordingly. The cells were then harvested and fixed with 70% cold ethanol at −20°C overnight. The cells were mixed with 100 μl of RNase for 30 min at 37°C and incubated with 400 μl of propidium iodide (PI) solution for 30 min at 4°C (shed from light). Finally, the DNA content (as an indicator of cell cycle distribution) was measured using a flow cytometer.

### 2.9 Flow cytometry analysis of cell apoptosis

We used flow cytometry to analyze cell apoptosis. First, the cells were incubated, divided into groups, and treated accordingly. An adequate number of cells were harvested, centrifuged, and resuspended in 300 µl of binding solution (approximately 1 × 10^6^ cells/ml). Annexin V‐FITC staining solution (4 µl) and PI solution (4 µl) were subsequently added to the binding solution. Finally, after the samples were placed on ice for 15 min, flow cytometry was conducted to detect apoptotic cells. The results were analyzed by FlowJo software (FlowJo v10.0.7).

### 2.10 Immunofluorescence staining of cells in vitro

Cell immunofluorescence staining was conducted to evaluate the expression of proteins related to angiogenesis and cell apoptosis. BMECs were plated in 12‐well plates (Corning) at a density of 1 × 10^5^ cells and treated as mentioned above. Then, the cells were fixed with 4% cold paraformaldehyde for 20 min, permeabilized with 0.5% Triton X‐100 for 15 min, and blocked with 5% normal goat serum for 1 h. Then, the samples were incubated at 4°C overnight with diluted antibodies against proteins related to cellular angiogenesis (CD31, VEGFR2, and Claudin‐1) and cell apoptosis (Caspase‐3, Bcl‐2, and Bax). The next day, the samples were rewarmed for 30 min, incubated with secondary antibody for 1 h, and incubated with DAPI for nuclear staining. Finally, the optical density of the fluorescence signal was measured with a Slideview VS200 slide scanner (Olympus, Japan).

### 2.11 In vitro Western blotting experiment

We used western blotting to measure the expression of target proteins to confirm the results of immunofluorescence staining. BMECs were seeded in 6‐well plates, cultured overnight to allow attachment, and then treated as described above. We extracted total protein from whole‐cell lysates. The samples were then mixed with 5× loading buffer at a 1:4 (v/v) ratio and boiled at 100 °C for 5 min. The proteins were separated by molecular weight on sodium dodecyl sulfate polyacrylamide gel electrophoresis (SDS‐PAGE) gels (8%, 10%, and 12%) and then transferred onto polyvinylidene difluoride (PVDF) membranes, which were incubated with blocking solution for 1 h and then with the indicated primary antibodies specific for target proteins, including GAPDH, Bcl‐2, Caspase‐3, Bax, CD31, VEGFR2, and Claudin‐1 (Abcam, Cambridge, UK), overnight at 4 °C. The next day, the membranes were exposed to secondary antibodies for 1 h. Between each step, the membranes were rinsed three times with Tris‐buffered saline with 0.1% Tween 20 (TBST) for 15 min. Finally, we used an enhanced chemiluminescence detection system (Bio‐Rad) to analyze the expression of target proteins, and the gray values of the target bands were analyzed with ImageJ software.

### 2.12 Animal modeling and grouping

We constructed an mouse (C57BL/6J) model of SAH by perforating the internal carotid artery (ICA) with a prolene suture, as described by Santillan et al.^17^ In short, after anesthesia and implantation of an intracranial pressure (ICP) monitor, a 10‐mm midline longitudinal incision was made over the thyroid bone. The omohyoid and sternomastoid muscles were retracted to expose the common carotid artery (CCA) and external carotid artery (ECA). The ECA was dissected, a permanent ligature was tied on its distal segment, and a temporary 6‐0 Prolene ligature was tied on the proximal segment to obtain a flow‐free vessel segment. A special round‐headed 5‐0 prolene suture (17 mm, polypropylene, ETHALLOY^tm^) was introduced into the ECA and advanced in a retrograde fashion toward the carotid bifurcation into the ICA. Perforation of the blood vessels, which was indicated by a feeling of breakthrough, to establish an SAH model, and model establishment was confirmed by sudden fluctuation of ICP. To examine the treatment effects of tFNAs in vivo, mice were divided into the following three groups: (i) the sham group, in which a prolene suture was inserted into the ICA, but perforation was not performed; (ii) the SAH group, in which the SAH model was constructed but tFNAs were not administered; and (iii) the SAH + tFNA treatment group, in which tFNAs were administered for 3 days after the establishment of the SAH model. All animals were evaluated in the same way. The protocol and procedures employed were ethically reviewed and approved by the institutional review board of West China Hospital, Sichuan University (2021245A).

### 2.13 Sample collection and histological examination

After surgery and treatment, the mice were anesthetized by i.p. injection of 10% chloral hydrate solution and subsequently transcardially perfused with PBS followed by 4% cold paraformaldehyde. The brains were extracted, fixed in 4% paraformaldehyde, dehydrated, embedded in paraffin, and sliced into 4‐μm brain sections. H&E staining was performed to observe vascular morphology, and immunohistochemical staining with the primary antibodies mentioned in the in vitro experiment section was conducted to visualize the expression of related proteins in vivo.

### 2.14 TEM

Tissues containing microvascular vessels were isolated from the dissected brains and cut into 1 × 1 × 1 mm^3^ pieces. The samples were fixed in 2.5% glutaraldehyde overnight, postfixed in 1% osmium tetroxide, dehydrated in acetone at different concentrations, and embedded in epoxy resin. Subsequently, the resin blocks were cut into 60‐nm pieces and exposed to uranyl acetate and lead citrate for observation. Images were acquired by TEM at an accelerating voltage of 80 kV, and the morphological characteristics of BMECs were quantified and analyzed.

### 2.15 In vivo Western blotting experiment

Microvascular tissue was isolated from the dissected mouse brains, and protein was extracted. The tissues were fixed with 4% paraformaldehyde and incubated in permeabilization solution. The extracted proteins were probed with the indicated primary antibodies, including antibodies against functional proteins (CD31, VEGFR2, and Claudin‐1) and proteins related to the PI3K‐AKT‐mTOR signaling pathway (P‐PI3K/PI3K, P‐AKT/AKT, and P‐mTOR/mTOR). The density of each band was measured with an ECL detection system.

### 2.16 Statistical analysis

Quantitative results are presented as the means ± standard deviations (SDs) of at least three. Intergroup differences were analyzed by one‐way analysis of variance (ANOVA) with GraphPad Prism v8.2.1 software. A two‐tailed *p* value <0.05 (*), <0.01 (**), or <0.001 (***) was considered.

## 3 RESULTS

### 3.1 Successful synthesis of tFNAs and uptake of tFNAs by BMECs

**FIGURE 1:**
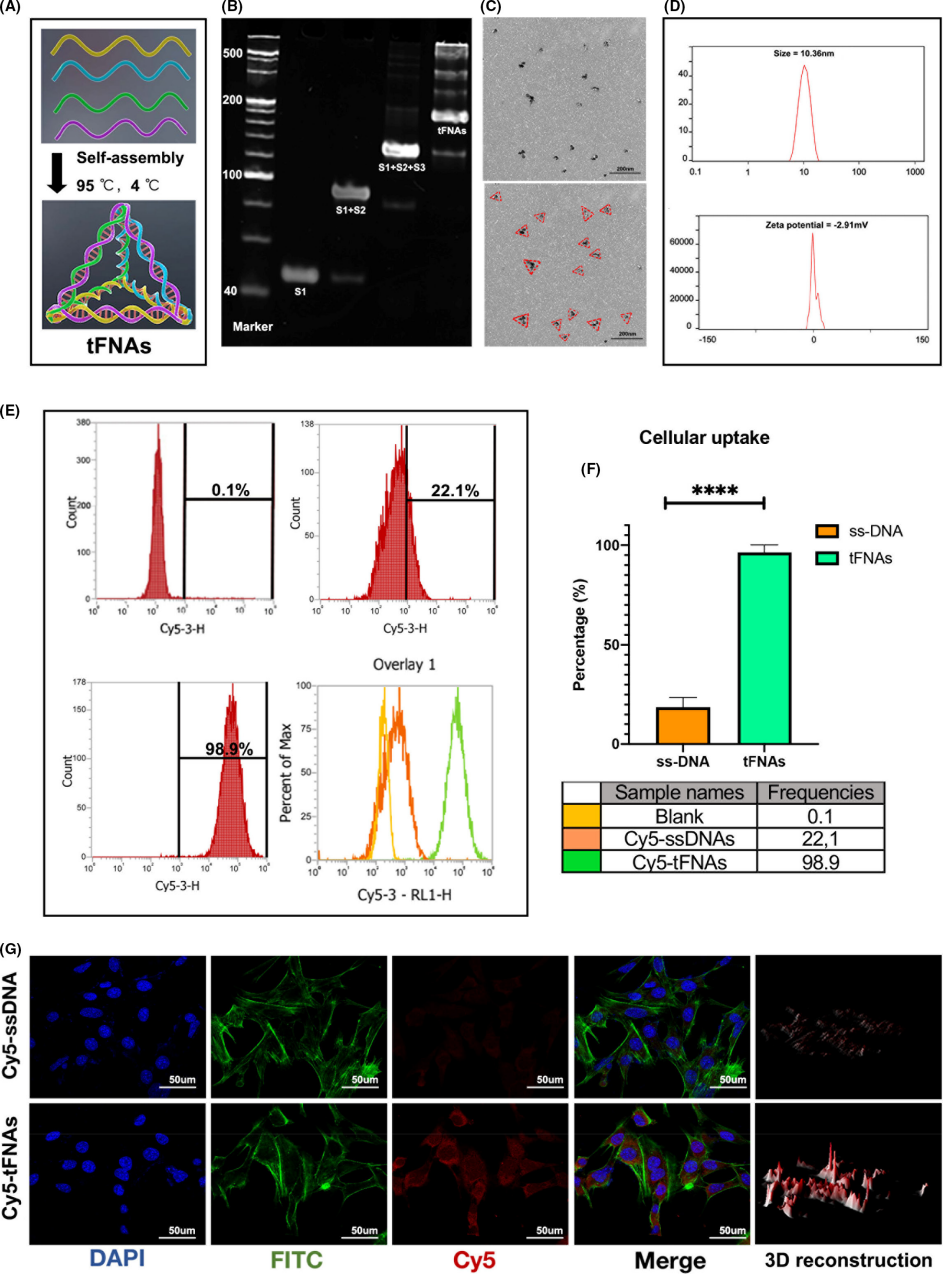
Successful synthesis and cellular uptake of tFNAs. (A) Schematic diagram of tFNAs. (B) 8% PAGE to confirm a successful fabrication of tFNAs. (C) Morphology and size of tFNAs by TEM (red triangle). Scale bars are 200 nm. (D) Size and Zeta potential distribution of the tFNA molecule. (E, F) Cellular uptake of Cy5‐tFNAs and Cy5‐ssDNAs by flow cytometry and statistical analysis. ****p < 0.0001. (G) Immunofluorescent images of Cy5 staining in BMECs (Cy5: red, nucleus: blue, cytoskeleton: green). Scale bars are 50 μm

As aforementioned, tFNAs were synthesized from four ssDNAs. Each ssDNA molecule was further divided into three small fragments, which were hybridized with each of the other three ssDNA fragments to form a tetrahedral structure through complementary base pairing (Figure 1A). To confirm the successful synthesis of tFNAs, PAGE was performed to determine the molecular weights of the tFNAs and ssDNAs (Figure 1B). In addition, tFNA morphology and particle size were assessed by TEM, and the results showed that there were some triangular nanoparticles with a size of approximately 10 nm (Figure 1C). The DLS results were consistent with the TEM results in terms of tFNA size. Additionally, zeta potential measurement proved that the tFNA molecules were negatively charged (Figure 1D).

We then used flow cytometry and immunofluorescence staining to evaluate the uptake of tFNAs and ssDNAs by BMECs (Figure 1G). Flow cytometry showed that tFNAs were taken up by BMECs and that the uptake rate of tFNAs was significantly higher than that of ssDNAs (Figure 1E,F). Similarly, immunofluorescence staining confirmed that the Cy5 fluorescence intensity in cells treated with tFNAs was much stronger than that in cells treated with ssDNAs.

### 3.2 Effects of tFNAs on cytotoxicity induced by hemin in vitro

Next, we focused on exploring the effects of tFNAs on cytotoxicity induced by hemin in vitro. As the main function of BMECs, angiogenesis refers to the complex process by which new blood vessels are constructed and involves cell activation, proliferation, and migration. Several cellular function assays, including cell viability assays, wound healing assays, transwell assays, tube formation assays, and flow cytometry for cell circle and apoptosis, were conducted.

**FIGURE 2:**
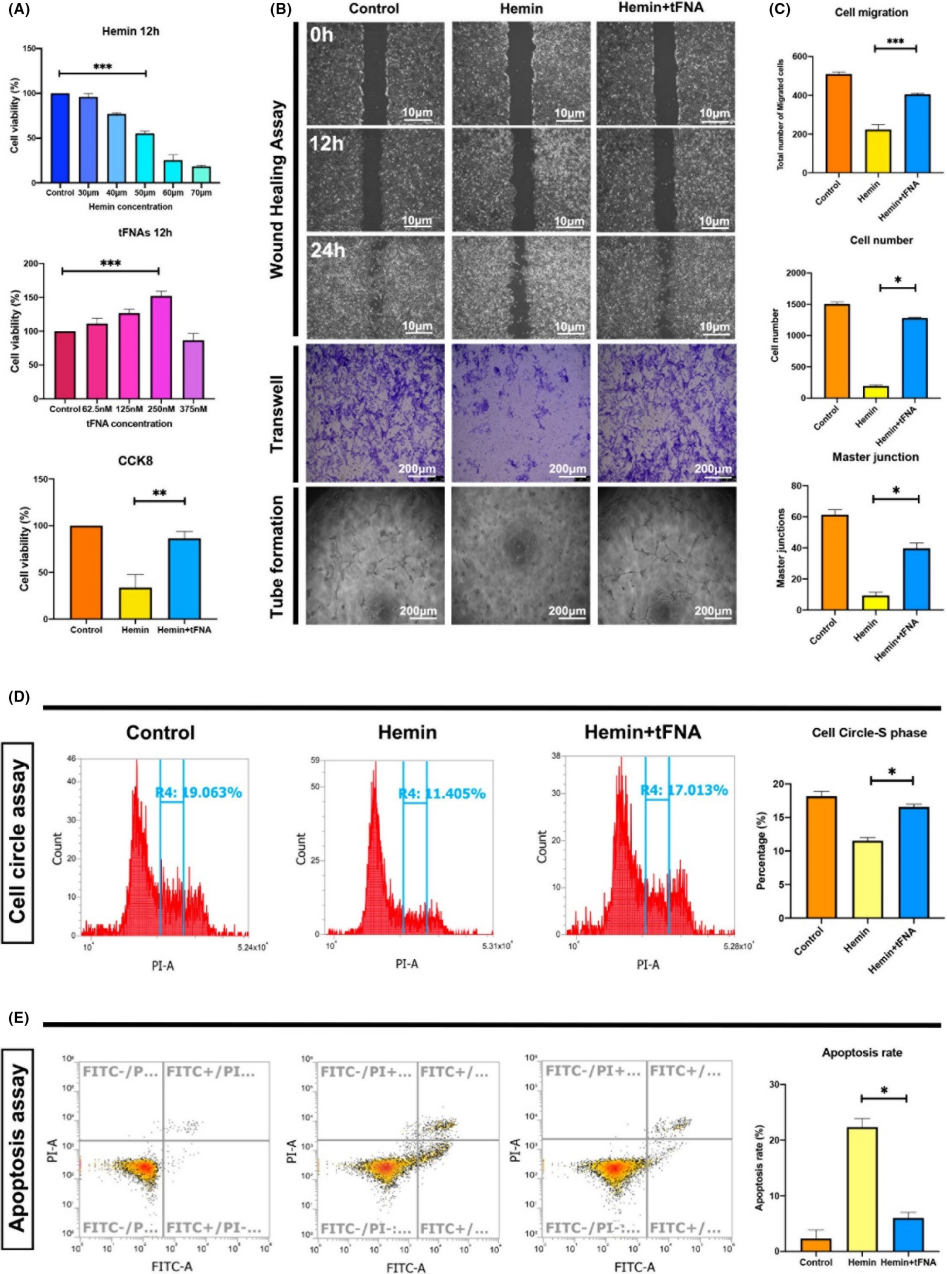
Effects of tFNAs on cytotoxicity induced by hemin. (A) The effects of tFNAs on hemin‐induced cell proliferation by cell viability essay. *p < 0.05, **p < 0.01, ***p < 0.001. (B) The effects of tFNAs on hemin‐induced cell migration and angiogenesis by wound healing assay, transwell assay and tube formation assay. Scale bars are 10 and 200 μm. (C) The statistical analysis of wound healing assay, transwell assay, and tube formation assay. *p < 0.05, **p < 0.01, ***p < 0.001. (D) The effects of tFNAs on hemin‐induced cell circle change by flow cytometry and statistical analysis. *p < 0.05. (E) The effects of tFNAs on hemin‐induced cell apoptosis by flow cytometry and statistical analysis. *p < 0.05

We used a cell viability assay to evaluate the cytotoxic effect of hemin at different concentrations on BMECs for 12 h. The results showed that the cell density was significantly reduced by hemin in a dose‐dependent manner, with an IC50 of approximately 50 ng/ml. Therefore, this concentration was selected for subsequent studies. Through the same approach, we determined the optimal concentration of tFNAs for promoting cell proliferation at a time point of 12 h, and the result was consistent with previous studies. tFNAs had the most beneficial effect on cell proliferation at a concentration of 250 nM but exhibited inhibited proliferation when the concentration reached 500 nM. To further demonstrate that tFNAs can reverse injury to BMECs caused by hemin, we sequentially treated cells with hemin (50 nM) and tFNAs (250 nM) for 12 h each. The results confirmed that the cell number significantly decreased upon exposed to hemin but was dramatically recovered after the administration of tFNAs (Figure 2A).

To assess cell migration ability, wound healing test (scratch test) and Transwell assay were conducted. The results revealed a similar trend: cell migration was significantly inhibited upon exposure to hemin but obviously promoted and more like that in the control group after treatment with tFNAs. Furthermore, we conducted a tube formation assay to assess the BMEC angiogenesis. Capillary tube formation was significantly reduced in cells exposed to hemin compared with control cells. However, the number of tube junctions was significantly increased when tFNAs were added to the culture medium (Figure 2B,C).

The cell cycle was analyzed by flow cytometry. S phase of the cell cycle plays an important role in DNA replication and therefore cell proliferation. There were significant differences in cell cycle distribution between the control and two experimental groups. Compared with the control group, the hemin group had a significantly lower percentage of cells in S phase. However, in cells treated with tFNAs, the percentage of cells in S phase was significantly increased and was more similar to that in the control group (Figure 2D).

The percentage of apoptotic cells was further evaluated by flow cytometry. The percentage of apoptotic cells was significantly elevated in the hemin group compared with the control group but significantly decreased in the hemin + tFNA group (Figure 2E).

### 3.3 Effects of tFNAs on the expression of cellular function‐related proteins and cell apoptotic‐related proteins in vitro

**FIGURE 3:**
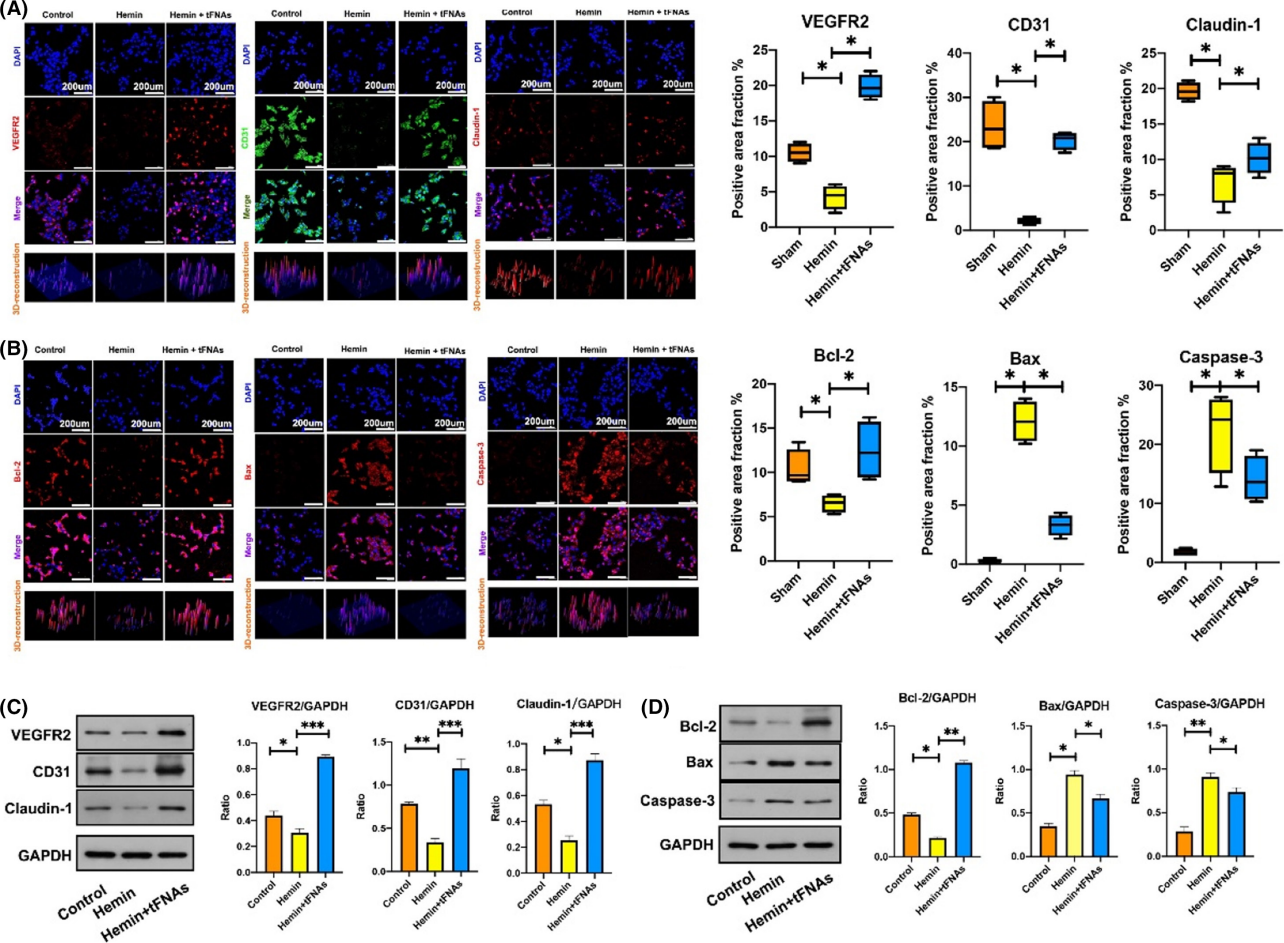
Effects of tFNAs on the expression of cellular function‐related proteins and cell apoptotic‐related proteins induced by hemin. (A) Representative immunofluorescent images with statistical analysis of function‐related proteins (VEGFR2, CD31, and Claudin‐1) in BMECs from three treatment groups (VEGFR2, Claudin‐1: red, CD31: green, nucleus: blue). Scale bars are 200 μm. *p < 0.05. (B) Representative immunofluorescent images with statistical analysis of apoptosis‐related proteins (Bcl‐2, Bax and Caspase‐3) staining in BMECs from three treatment groups (Bcl‐2, Bax and Caspase‐3: red, nucleus: blue). Scale bars are 200 μm. *p < 0.05. (C) Western blot with statistical analysis of the expression levels of VEGFR2, CD31, and Claudin‐1 from three treatment groups. *p < 0.05, **p < 0.01, ***p < 0.001. (D) Western blot with statistical analysis of the expression levels of Bcl‐2, Bax, and Caspase‐3 from three treatment groups. *p < 0.05, **p < 0.01, ***p < 0.001

The expression of proteins related to BMEC functions, specifically the regulation of angiogenesis, such as VEGFR2, CD31, and Claudin‐1, was assessed by immunofluorescence staining to explore the effect of tFNAs on the functional recovery of injured BMECs induced by hemin. The results showed that the fluorescent signals of these three functional proteins were dramatically decreased in cells exposed to hemin, while the signals were significantly increased and more similar to those in the control group upon treatment with tFNAs (Figure 3A). We then evaluated the expression of various apoptosis‐related proteins, including Bcl‐2, Bax, and Caspase‐3, by immunofluorescence staining to explore the mechanism by which tFNAs inhibit BMEC apoptosis induced by hemin. The results showed the expression of the apoptosis mediators Bax and Caspase‐3 were significantly decreased, while the expression of the anti‐apoptotic protein Bcl‐2 was significantly increased in the hemin + tFNA group compared with hemin group (Figure 3B). Notably, the results of western blot analysis of the expression of these function‐related proteins and cell apoptotic‐related proteins were consistent with the results of immunofluorescence staining (Figure 3C–D). Together, these in vitro results suggested that tFNA treatment suppressed angiogenesis of injured BMECs by suppressing hemin‐induced apoptosis.

### 3.4 Effect of tFNAs on SAH‐induced diffuse BMEC injury in vivo

**FIGURE 4:**
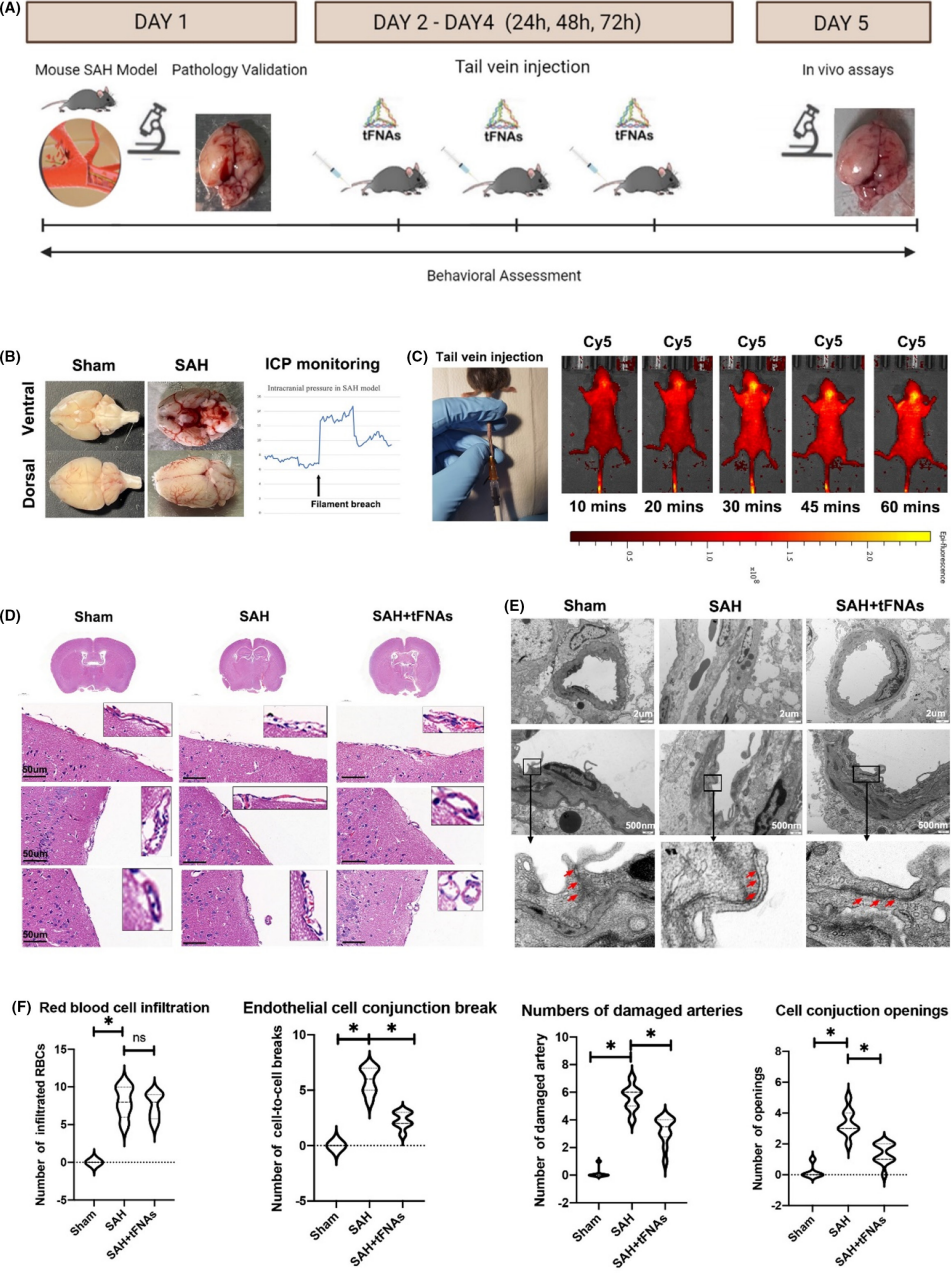
Effects of tFNAs on SAH‐induced diffuse BMEC injury. (A) A schematic drawing of the animal experiment design in SAM model. The experimental groups were designed as follows: Sham group (a prolene suture was inserted into the ICA but perforation was not performed), SAH group (SAH model was constructed), tFNA treatment group (tFNAs were administered for 3 days after the establishment of the SAH model). (B) Obvious SAH on the surface of the brain tissue in the anatomical specimens and a sharp increase in ICP indicated a successful animal model establishment. (C) Live fluorescence images of the in vivo distribution of tFNA‐Cy5 after tail vein injection. (D) Representative H&E staining images for microvascular bed tissue from three treatment groups. Scale bars are 50 μm. (E) Representative TEM images for microvascular bed from three treatment groups. Scale bars are 2 μm and 500 nm. (F) Statistical analysis of H&E and TEM. *p < 0.05

The animal experiment protocol is shown in Figure 4A. To confirm the effect of tFNAs on BMEC injury after SAH in vivo, a mouse model of SAH was established through surgical perforation of the ICA with a Prolene suture as described above. Successful modeling was indicated by a sharp increase in ICP and obvious SAH on the surface of the brain tissue in the anatomical specimens (Figure 4B). Upon establishment of the SAH model, the mice were administered saline or tFNAs for three days. In vivo imaging analysis showed that when administered via tail vein injection, tFNAs remained in the brain vascular system and maintained a strong fluorescence signal for at least 60 min (Figure 4C).

The effects of tFNAs on SAH‐induced diffuse BMEC injury were explored by histological examination and TEM. H&E staining was conducted to compare the general morphology of the microvascular bed on the surface of brain tissue among the three groups of mice as mentioned above. The results showed normal artery contour and BMEC lineage in sham group. BMECs were intact and continuously connected in the tunica intima of brain surface arteries. No red blood cells or myeloid‐derived inflammatory cells were observed infiltrated in or adjacent to the arteries. In SAH group, a reduced BMEC number was noted compared with the sham group. Distribution of BMECs was also sparser and discontinuous, indicating unneglectable acute injury to endothelial cells in the microvascular bed. Notably, in the tFNA treated group, despite the damage resulted from initial SAH injury, microvascular bed morphology was more intact compared to single SAH model. Endothelial cells were more closely connected, maintaining the part of tunica intima integrity in brain surface arteries (Figure 4D).

TEM revealed more details about the micro‐angioarchitecture differences in BMECs among the three groups. Briefly, TEM results corroborated with H&E staining observations, indicating more obvious microvascular bed injury in the SAH group and alleviated endothelial damage upon treatment with tFNAs. In detail, TEM showed the intact and continuous BMEC‐to‐BMEC conjunctions in sham group were significantly more opened in SAH group (pointed by red arrows in Figure 4E). While in tFNAs treated group, the damage was clearly attenuated, exhibiting more intact endothelial cellular morphology and alleviated cell‐to‐cell conjunction opening gaps (Figure 4E) (Figure 4E).

We statistically analyzed the SAH damage by comparing the infiltration of red blood cells, breaks of endothelial cell conjunctions, and numbers of damaged arteries based on H&E staining. We noticed similar increased red blood cell infiltration in SAH and SAH + tFNAs group, suggesting similar hemorrhage damage in two groups. Endothelial cells observation indicated more conjunctions breaks at cellular interfaces in SAH group but was lowered in SAH + tFNAs group. In whole brain atlas, significantly more damaged arteries were also observed in SAH group, compared to sham and SAH + tFNAs. In TEM, we noticed significantly wider cell‐to‐cell junction gap openings in SAH. SAH + tFNAs showed alleviated damage after tFNAs intravenous injection (Figure 4F). In all, H&E and TEM results jointly indicated that the tFNAs attenuate the damage caused by SAH in situ, showing restored number of BMECs in the endothelium layer and more tight intercellular connectivity.

### 3.5 Effects of tFNAs on the expression of cellular function‐related proteins and cell apoptotic‐related proteins in vivo

**FIGURE 5:**
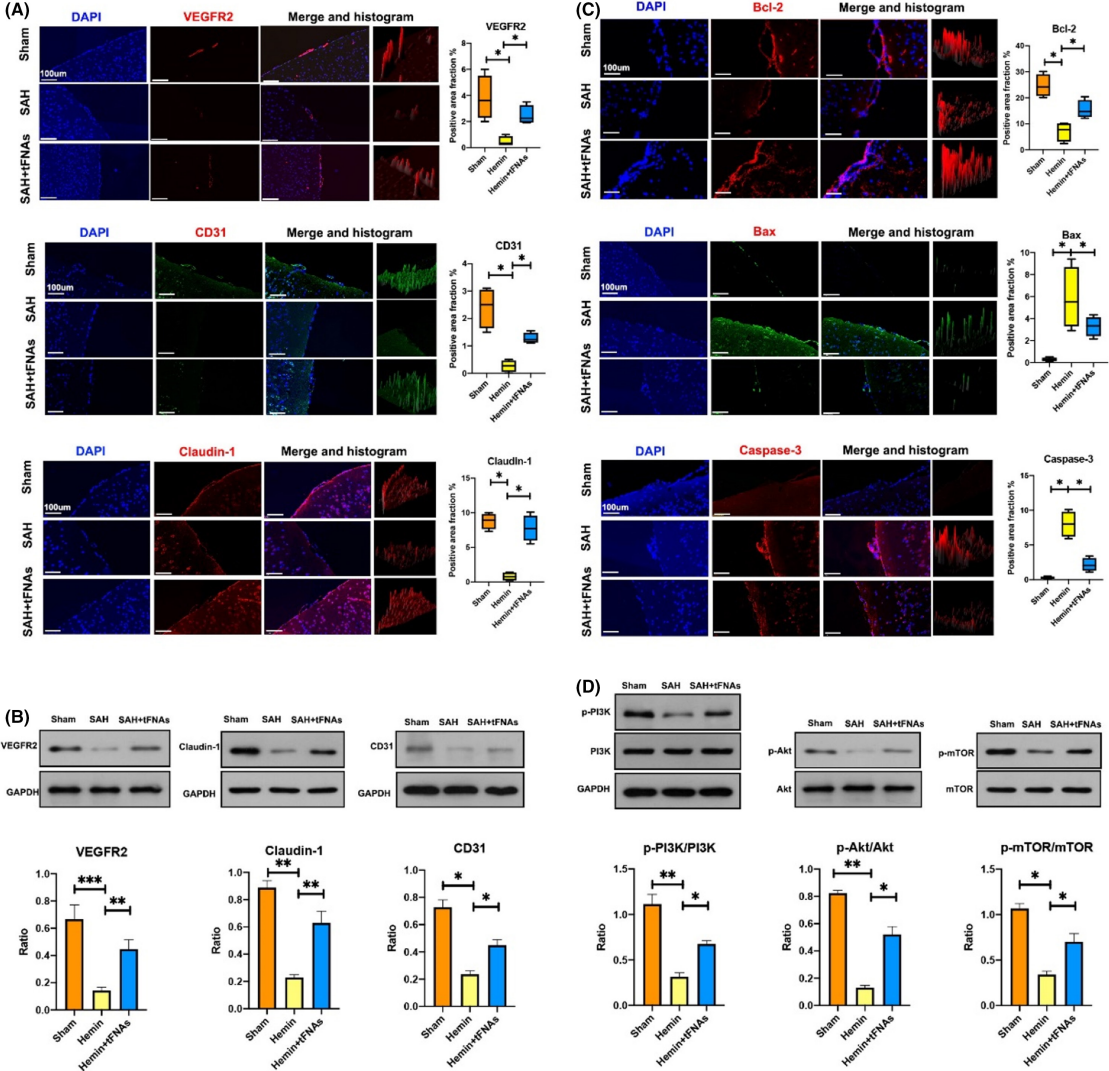
Effects of tFNAs on the changes in related proteins in the microvascular bed and underlying mechanisms. (A) Representative immunofluorescent images with statistical analysis of function‐related proteins (VEGFR2, CD31, Claudin‐1) staining in the microvascular bed from three treatment groups (VEGFR2, Claudin‐1: red, CD31: green, nucleus: blue). Scale bars are 100 μm. Data are presented as mean ± SD (*n* = 3), *p < 0.05. (B) Western blot with statistical analyses of VEGFR2, CD31, Claudin‐1 from three treatment groups. Data are presented as mean ± SD (*n* = 3). *p < 0.05, **p < 0.01, ***p < 0.001. (C) Representative immunofluorescent images with statistical analysis of apoptosis‐related proteins (Bcl‐2, Bax and Caspase‐3) staining in the microvascular bed from three treatment groups (Bcl‐2, Bax and Caspase‐3: red, nucleus: blue). Scale bars are 100 μm. Data are presented as mean ± SD (*n* = 3), *p < 0.05. (D) Western blot with statistical analysis of PI3K‐AKT‐mTOR pathway‐related proteins and quantification of the phosphorylation level of PI3K, AKT, and mTOR in microvascular bed from three treatment groups. Data are presented as mean ± SD (*n* = 3). Statistical analysis: *p < 0.05, **p < 0.01

We assessed the expression of three functional proteins (VEGFR2, CD31, and Claudin‐1) by immunofluorescence staining of brain sections (Figure 5A) and western blotting (Figure 5B). The results showed a similar trend to that observed in vitro. Compared with the sham group, the microvascular bed in SAH model group had significantly lower expression of these three proteins. However, upon tFNA treatment, the expression of the three proteins was significantly increased.

To confirm the antiapoptotic effect of tFNAs on injured BMECs in vivo, immunohistochemical staining of brain sections was conducted using different primary antibodies against apoptotic‐related proteins (Bcl‐2, Bax, and Caspase‐3). The results were consistent with the above‐mentioned in vitro results. Compared with that in the sham group, the expression of the apoptosis mediators Bax and Caspase‐3 in the microvascular bed of SAH model group was significantly increased. The expression of the antiapoptotic protein Bcl‐2 was significantly decreased. In the SAH + tFNA group, the expression of Bax and Caspase‐3 was significantly decreased, and the expression of Bcl‐2 was largely increased. The expression levels in the SAH + tFNA group were more similar to those in the sham group (Figure 5C).

### 3.6 Effects of tFNAs on the PI3K‐AKT‐mTOR signaling pathway in vivo

To investigate whether the antiapoptotic effect of tFNA on injured BMECs is related to the PI3K‐AKT‐mTOR signaling pathway, we conducted western blot analysis of protein expression in brain microvascular tissue from the different groups as mentioned above. The results showed that when normalized to GAPDH expression, the ratios of P‐PI3K/PI3K, P‐AKT/AKT, and P‐mTOR/mTOR were significantly decreased in the SAH group but were increased and more similar to those in the sham group upon treatment with tFNAs (Figure 5D).

## 4 DISCUSSION

According to previous epidemiological reports, aneurysmal SAH has an annual incidence of 6–27.63 per 100,000 persons in people of all ages and accounts for 5–7% of all strokes.^18^, ^19^ Despite improvements in neurocritical care management in recent decades, the mortality rate of patients with SAH remains up to 40–50%. Among those who survive, only two‐thirds regain functional independence 1 year after hemorrhage.^20^, ^21^ One major reason is that, unlike cerebral hematoma occur in the brain parenchyma, SAH leads to extensive distribution of blood on the surface of brain cortex where sits the monolayer brain vascular endothelial cells in the microvascular bed.^22^ Damage of BMECs play can result in dysregulation and disrupted substance exchange between blood and brain tissues.^23^ Diffuse injury to BMECs is a trigger for several fatal pathophysiological consequences after SAH and is related to a poor prognosis.^24^ However, there is an urge in practice to discover an effective treatment for diffuse BMEC injury after SAH.

In our study, we explored the treatment effect of tFNAs on diffuse injury to BMECs after SAH. In consistent with previous discoveries, DNA nanomaterials also exhibit excellent editability and biological applications among similar materials.^9^ Our results corroborated with the regulatory effect of tFNAs in cellular biological functions such as migration, proliferation, differentiation, and autophagy and further suggest potent biological functions with practical importance for critically injured terminally differentiated cells such as BMECs.^11^, ^12^, ^13^

We first confirmed in different ways that tFNAs were successfully synthesized and taken up by cells, which was the foundation for our subsequent functional experiments. Next, we focused on exploring the effects of tFNAs on cytotoxicity induced by hemin in vitro. As the main function of BMECs, angiogenesis refers to the complex process by which new blood vessels are constructed and involves cell activation, proliferation, and migration. This process is closely related to cell viability. Several cellular functional assays, including cell viability assays, wound healing assays, transwell assays, and tube formation assays, were conducted, and the results confirmed that decreases in cell proliferation, migration, and tube formation ability could be significantly inhibited by treatment with tFNAs. In addition, immunofluorescence staining and western blotting revealed that the expression of cellular function‐related proteins, including VEGFR2, CD31, and Claudin‐1, was dramatically decreased upon exposure to hemin, but this change in expression was significantly reversed by treatment with tFNAs. This treatment effect might be related to changes in the expression of mitochondrial apoptosis‐related proteins, such as Bcl‐2, Bax, and Caspase‐3, as indicated by cell immunofluorescence staining and western blotting. These results suggested that tFNA treatment suppressed angiogenesis of injured BMECs by suppressing hemin‐induced apoptosis.

Worth noticing, we chose hemin in our in vitro experiment to simulate the effect of SAH due to its high clinical relevance. Among the biomolecules reported by previous research, hemin, a breakdown product of hemoglobin (Hb), is one of the key factors related to diffuse injury to BMECs.^25^ Following the expansion of hemorrhage after SAH, Hb in red blood cells is released into the subarachnoid space within hours to days and is metabolized into hemin. The released hemin is subsequently metabolized into carbon monoxide, biliverdin, and iron, which are gradually released into surrounding tissues, including the brain microvascular bed, and affect their normal functions.^26^ It has been reported that hemin has a detrimental effect on BMECs through multiple processes, including the release of redox‐active iron, depletion of cellular stores of NADPH and glutathione, the production of superoxide and hydroxyl radicals, and peroxidation of membrane lipids, which leads to the disruption of cell junction integrity and cell apoptosis.^27^ Therefore, hemin was selected as model molecule in this present research.

In the in vivo animal experiment, a mouse model of SAH was constructed by puncture of the ICA with a prolene suture. Model establishment was confirmed by a sudden fluctuation in ICP monitor and bleeding on the surface of brain tissue specimens. This model was chosen owing to its highly identical nature to the spontaneous hemorrhage induced by aneurysm rupture. Other models were considered but excluded like the injection of autologous blood to the basal cistern. Because the injection model should be better adopted in studying epilepsy or brain parenchyma damage after SAH.

For histological examination, H&E staining and TEM were performed using SAH rodent model, and the results suggested that a diffuse injury of BMEC in the microvascular bed was induced by SAH which was successfully reversed by treatment with tFNAs. In addition, similar to the in vitro results, the expression of angiogenesis‐related proteins (VEGFR2, CD31, and Claudin‐1) in mouse brain sections was significantly decreased in the SAH model group and recovered by treatment with tFNAs. Moreover, the change in the expression of apoptosis‐related proteins (Bcl‐2, Bax, and Caspase‐3) was consistent with the trends reported in vitro. Together, these results suggest the therapeutic potential of tFNA in the treatment of diffuse BMEC injury in vivo after SAH.

In an effort to better understand the molecular and cellular mechanisms underlying the antiapoptotic and proangiogenic effects of tFNAs in the treatment of BMEC injury, we attempted to study the PI3K‐AKT‐mTOR signaling pathway, a crucial signaling pathway that can regulate many cellular functions, including proliferation, adhesion, migration, invasion, metabolism, and survival via phosphorylation.^28^ PI3K‐AKT‐mTOR signaling pathway participates in angiogenesis during physiological development and under pathological conditions in several disease models.^29^, ^30^ Our present study shows similar results with previous conclusions that activation of the PI3K/AKT pathway can not only increase the production of CD31, a biomarker of blood vessels, but also modulate the expression of other angiogenic factors, such as VEGF and Claudin‐1/4.^31^, ^32^, ^33^ In addition, sustained activation of this pathway in endothelial cells has been shown to induce the abnormal formation of blood vessels that recapitulate the aberrations of tumor vessels, and inhibition of this signaling pathway is one of the key strategies for controlling angiogenesis in the treatment of cancer.^34^, ^35^, ^36^, ^37^

Western blot analysis of the expression of proteins related to the PI3K‐AKT‐mTOR signaling pathways in vivo confirmed that tFNAs might promote cell recovery and angiogenesis through this signaling pathway. Previous study has reported PI3K‐AKT‐mTOR is a crucial signaling pathway, which can promote cellular survival through inhibition of apoptotic factors and activation of anti‐apoptotic factors.^38^, ^39^, ^40^ The function of the antiapoptotic protein Bcl‐2 and the proapoptotic protein Bax can be regulated in an AKT‐dependent manner. Activation of AKT by phosphorylation at Ser136 and Ser184 promotes the function of Bcl‐2 and suppresses the activation of Bax, respectively.^41^ Changes in the expression of Bcl‐2 and Bax may be related to decreased permeability of the mitochondrial intermembrane, which can inhibit the release and mobilization of cytochrome c and consequently suppress the activation of the proapoptotic protein Caspase‐3.^42^, ^43^ Taken together, these data indicate that activation of the PI3K‐AKT‐mTOR signaling pathway was promoted by tFNAs via phosphorylation, which can suppress cell apoptosis and promote the expression of angiogenesis‐related proteins in injured BMECs after SAH, increase cell viability, and consequently increase angiogenesis.

## 5 CONCLUSIONS

Taken together, our results show that tFNAs, as novel and functional DNA nanomaterials, can significantly prevent hemin‐induced cytotoxicity to BMECs by regulating mitochondrial apoptotic pathways. In a mouse model of SAH, tFNAs can promote angiogenesis by increasing the expression of angiogenesis‐related proteins and inhibiting cell apoptosis in mouse brain microvascular tissue. These treatment effects might be associated with activation of the PI3K‐AKT‐mTOR signaling pathway, which is promoted by the tFNAs via an increase in phosphorylation. The results of the current study indicate the potential therapeutic effects of tFNAs on tissue vascularization, especially in the treatment of patients with diffuse BMEC injury after SAH.

## AUTHOR CONTRIBUTION

The authors confirm contribution to the paper as follows: study conception and design: Ruiqi Chen, Yunfeng Lin, Chao You; Experiment and data collection: Ruiqi Chen, Dingke Wen, Wei Fu, Lu Xing, Yi Liu, Hao Li; Analysis and interpretation of results:Ruiqi Chen, Dingke Wen, Lu Xing, Lu Ma, Yi Liu, Hao Li; Draft manuscript preparation:Ruiqi Chen, Dingke Wen, Lu Xing, Lu Ma. All authors reviewed the results and approved the final version of the manuscript.

## CONFLICT OF INTEREST

The authors declare no conflict of interest.

## Data Availability

The data that support the findings of this study are available from the corresponding author upon reasonable request.
